# The calculated versus the measured glycosylated haemoglobin (HbA_1c_) levels in patients with type 2 diabetes mellitus

**DOI:** 10.1002/jcla.23873

**Published:** 2021-06-14

**Authors:** Imad R. Musa, Saeed M. Omar, Manal E. Sharif, Abdel B. A. Ahmed, Ishag Adam

**Affiliations:** ^1^ Royal Commission Hospital in Al Jubail Industrial City Al Jaubil Saudi Arabia; ^2^ Faculty of Medicine Gadarif University Gadarif Sudan; ^3^ College of Medicine King Khalid University Abha Saudi Arabia; ^4^ Department of Obstetrics and Gynecology Unaizah College of Medicine and Medical Sciences Qassim University Unaizah Saudi Arabia

**Keywords:** calculated glycosylated haemoglobin A, diabetes mellitus, fasting blood sugar, Sudan

## Abstract

**Background:**

Diabetes mellitus (DM) is a chronic metabolic disorder that is increasing globally. It is associated with chronic complications that are more common among patients with poor glycaemic control. Glycosylated haemoglobin (HbA_1c_) is the gold standard for monitoring glycaemic control. Measurements of HbA_1c_ are relatively expensive and not available in some remote areas of developing countries.

**Methods:**

We conducted a cross‐sectional study to evaluate the agreement between the calculated and measured HbA_1c_ levels. The equation to compute the calculated HbA_1c_ also incorporated the fasting blood glucose (FBG) level and was as follows: HbA_1c_ = 2.6 + 0.03 × FBG (mg/dl).

**Result:**

We enrolled 290 patients with type 2 DM in this study. Of these, 204 (70.3%) were females and the mean (SD) age was 54.9 (12.8) years. The mean (SD) diabetes duration was 6.8 (5.5) years. There were 211 (72.8%) patients using oral hypoglycaemic agents, 62 (21.4%) were using insulin and 17 (5.9%) were using both insulin and oral hypoglycaemic agents. There was a borderline difference between the mean (SD) calculated and measured HbA_1c_ levels (*p* = 0.054). There was a significant correlation between the calculated and measured HbA_1c_ (*r* = 0.595, *p* < 0.001). However, there was no agreement between the calculated and measured HbA_1c_. The bias ±SD (limits of agreement) for calculated versus measured HbA_1c_ was −1.008 ± 2.02% (−5.05, 2.032).

**Conclusion:**

Despite the presence of a significant correlation between the calculated and measured HbA_1c_, the calculated level has shown an unacceptable agreement with the measured HbA_1c_.

## Graphical abstract

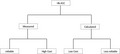

## 1 INTRODUCTION

Diabetes mellitus (DM) is a major health problem that has reached alarming levels, as nearly half a billion individuals live with DM worldwide. Type 2 DM comprises the vast majority (around 90%) of patients with DM globally.^1^ According to the International Diabetes Federation, the estimated global number of patients with DM was 463 million in 2019 and is expected to reach to 700 million by 2045, a 51% increase.^1^ The number of patients with DM is estimated to increase by 143% in Africa in 2019, and the number of individuals with DM was 19 million and estimated to jump to 47 million by 2045.^1^ These data underscore the huge burden DM will present for the future in developing countries if no efforts are taken to control it. Individuals with DM are at a high risk of serious complications (in particular those with type 2 DM), such as heart disease, renal disease, stroke, atherosclerosis, peripheral neuropathy and blindness.^1^ Monitoring glycaemic control among diabetic patients is of paramount importance to modify the prognosis and the outcome.^1^, ^2^ The glycated haemoglobin A_1c_ (HbA_1c_) and fasting blood glucose (FBG) levels are important tools for the diagnosis and monitoring of glycaemic control with a cut‐off of 6.5% and 126 mg/dl, respectively.^1^, ^3^ The role for a mathematical formula to calculate HbA_1c_ has gained interest since it is a simple and cost‐effective method.^4^ Basically, glycated haemoglobin is a form of haemoglobin used primarily to estimate the average plasma glucose concentrations over prolonged periods of three to four months.^5^ Hence, it is promoted for monitoring glycaemic control in patients with DM as well as a method for diagnosing DM.^6^, ^7^ In developing countries, where there are several resource‐poor settings and HbA_1c_ test availability is a concern, calculations for HbA_1c_ may offer hope and help.^4^ Moreover, the estimated HbA_1c_ can be a reasonable means to reduce the financial burden, particularly in developing countries.^4^, ^5^, ^8^ In fact, the health expenditure in Africa is relatively low, with the mean health expenditure per person with diabetes in Africa being less than 400 U.S. dollars, compared to 6,800 U.S. dollars in North America and the Caribbean nations.^1^ The prevalence of DM in Sudan was 22.1% in 2019, and it is estimated to reach 24.2% by 2045.^1^ This is in accordance with a recently published study from Sudan.^9^, ^10^ Sudan is not exempt when it comes to having limited healthcare resources and facilities. Most of the populations are not covered by a health insurance system, and there is a high cost for health services as well as limited availability for HbA_1c_ measurements in most rural areas. Moreover, some recent studies from Sudan have demonstrated a high prevalence of DM and uncontrolled DM.^9^, ^10^ Hence, we conducted this study to evaluate the agreement between the calculated and measured HbA_1c_ among patients with type 2 DM.

## 2 METHODS

### 2.1 Study design and ethic approval

This was cross‐sectional study conducted between April and October 2019. Patients were recruited from the outpatient diabetic/endocrine clinic at Gadarif Teaching Hospital. We recruited all patients with type 2 DM. This included both males and females, aged 18 years or older and those with haemoglobin levels between 12 and 16 g/dl because HbA_1c_ results can be affected by several factors, including anaemia. The exclusion criteria were patients with type 1 DM, haemoglobinopathies, functional thyroid disorders, patients with hypertension and on diuretics, renal disorders, anaemia (haemoglobin ˂12 g/dl), bedridden patients, patients with an advanced malignancy and pregnant women.

The data were collected using a standardized questionnaire that included demographic data (age and sex), comorbidities, weight, height, haemoglobin level, FBG, measured HbA_1c_ and calculated HbA_1c_ levels based on the adopted equation.

Universal safety precautions were adopted for collecting the blood samples. Sterile disposable needles and vacutainers were used to obtain the samples. Correct procedures were adopted at all steps and included the venepuncture site and the pressure used to transfer blood into the vacutainer to prevent haemolysis. After signing a written informed consent form, about 4 ml of venous blood was drawn under aseptic conditions in ethylenediaminetetraacetic acid (EDTA) lavender top and sodium fluoride‐containing grey top vacutainers and processed accordingly. Grey top vacutainers were centrifuged at 3000 rpm for 15 min, and the sample was obtained.

1. The plasma glucose levels were measured by the glucose oxidase‐peroxidase method as per the manufacturer's instructions (Shino‐Test Corp.)
2. The EDTA samples were analysed in a Mispa *i3* auto analyser by nephelometry (Agappe Diagnostics Switzerland GmbH) to obtain the measured evaluated agreement between the calculated and measured HbA_1c_ levels.^11^
3. The American Diabetes Association (ADA) reference ranges were adopted as follows: ˂5.7% (non‐diabetic range); 5.7%–6.4% (high‐risk group); and ≥6.5% (diabetics).^12^
4. The equation used to assess the calculated HbA_1c_ is based on the FBG as follows: Calculated HbA_1c_ = 2.6 + 0.03 × FBG (mg/dl).^7^
5. The patients’ weights and heights were measured using standard procedures, and body mass index (BMI) was computed as weight (kg)/height (m^2^).

Guided by Bujang and Baharum,^13^ a sample of 290 patients with type 2 DM was calculated to have the significant minimum difference in the correlations (*r *= 0.15) for calculated HbA_1c_ and measured HbA_1c_. This sample would have an 80% power and a difference of 5% at *α* = .05.

### 2.2 Statistical analysis

Data were analysed using the Statistical Package for Social Sciences (SPSS) version 22 software (SPSS Inc.). Descriptive data were represented as the mean and standard deviation (SD). Pearson correlation analyses were performed to determine the correlation coefficient (*r*) between the calculated and measured HbA_1c_. The bias (mean of the differences and SD) and limits of agreement (mean ±2 SD) were calculated as previously described by Bland and Altman.^14^ Limits of the agreement not exceeding ±1% were considered clinically acceptable.

### 2.3 Ethics

This study received ethical approval from the Research Board at the Faculty of Medicine, University of Gadarif, Sudan. The reference number is 2019/38. Written informed consent was obtained from all the enrolled patients.

## 3 RESULTS

There were 290 patients enrolled in the study. Of these, 204 (70.3%) were females. The mean (SD) age was 54.9 (12.7) years. The mean (SD) diabetes duration was 6.8 (5.5) years. There were 211 (72.8%) patients were using oral glucose control agents, 62 (21.4%) patients were using insulin and 17 (5.9%) patients were using both insulin and oral glucose control agents (Table 1).

**TABLE 1:** General characteristic of the enrolled patients

Variables	Mean	Standard deviation
Age (years)	54.8	12.8
Males	102	30.1
Education ≤secondary level	259	76.4
Married	305	90.0
Employed	150	44.2
Duration of diabetes (years)	6.8	5.5
Fasting blood glucose (mg/dl)	163.7	64.8
Haemoglobin A_1_ (%)	8.6	2.1

**FIGURE 1:**
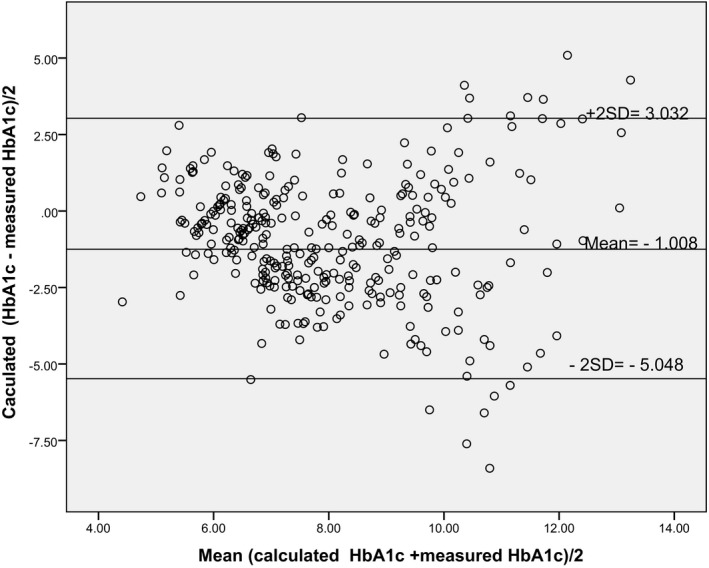
The Bland‐Altman plot for the calculated versus the measured HBA_1c_ levels

There was a borderline difference between the mean (SD) calculated and measured HbA_1c_ (*p* = 0.054) (Table 2). There was a significant correlation between the calculated and measured HbA_1c_ (*r* = 0.595, *p* < 0.001). Alternatively, there was no agreement between the calculated and measured HbA_1c_. The bias ±SD (limits of agreement) for the calculated versus measured HbA_1c_ was HbA_1c_ ‐ 1.008 ± 2.02% (₋5.05, 2.032) (Table 3, Figure 1).

**TABLE 2:** Measurement of the estimated and actual glycosylated haemoglobin (HbA_1c_) levels

Variables	Measured haemoglobin A_1_	Calculated haemoglobin A_1_
Mean	8.5	7.5
Median	8.5	7.0
Standard deviation	2.11	1.9
Range	4–15	2.9–15.3

**TABLE 3:** Correlation, bias and limits of agreement between the calculated and measured haemoglobin A_1c_ levels

Measurements	Correlation coefficient	Bias ±SD (95% confidence interval)	Limits of agreement
Values	0.595	−1.008 ± 2.02 (−1.22, −0.79)	−5.05, 2.032

## 4 DISCUSSION

The current study showed that, although there was a significant positive correlation between the calculated and measured HbA_1c_, there was an unacceptable agreement between the measured and calculated HbA_1c_. Thus, the non‐agreement obtained in our results pointed to an unreliability of the calculated HbA_1c_ using the equation to assess glycaemic control. Likewise, one study has shown a statistically significant negative bias among patients with or without DM when the Bland‐Altman plot was made.^5^ Moreover, they reported a non‐significant difference between the measured and calculated HbA_1c_ levels.^5^ They further explained the significant negative bias in patients with DM to hyperglycaemia in uncontrolled DM.^5^ Previously, Nayal et al. observed a significant difference between the erythrocyte HbA_1c_ levels and the calculated HbA_1c_ levels.^6^ In Sudan, it has recently been shown that HbA_1c_ has a poor reliability, insufficient sensitivity or specificity for diagnosing gestational DM.^3^

Previous studies have recommended the use of the calculated HbA_1c_ based on self‐measured glucose and past HbA_1c_ values for assessing glycaemic control in patients with DM, in particular among those with good glycaemic control.^6^, ^7^ HbA_1c_ is subject to variations, irrespective of the glycaemic control. Some of the putative factors for these variations include age, sex hormones, visceral fat distributions, physiologic and genetic factors and socioeconomic status.^15^, ^16^ The significant correlation between the calculated and measured HbA_1c_ which was obtained in our study may be explained by the observation that higher HbA_1c_ levels were seen in patients with persistently elevated blood glucose levels, particularly in patients with uncontrolled DM.^5^, ^17^ Additionally, those with good glycaemic control had HbA_1c_ levels close to, or within, the reference range, which might provide identical values using the same mathematical formula (HbA_1c_ = 2.6 + 0.03 × FBG [mg/dl]).^5^ Interestingly, another study adopting the same formula obtained a significant difference between the measured and calculated HbA_1c_ among patients with type 2 DM and a control group to assess glycaemic control.^4^ ThFBSey found that HbA_1c_ values derived and predicted by the formula were in accordance with measured values using the high‐performance liquid chromatography (HPLC) BIORAD method.^4^ Additionally, some studies have used other equations to estimate HbA_1c_ that were helpful in estimating glycaemic control based on a significant correlational difference.^17^, ^18^ Unfortunately, the limits of agreement were not assessed to consider it clinically acceptable. Likewise, one study has shown a significant difference between the levels of measured and calculated HbA_1c_ based on the calculated fasting blood glucose (FBG) (HbA_1c_ = 2.6 + 0.03 × blood glucose [mg/dl]).^19^ Interestingly, they found the calculated HbA_1c_ was not identical to the measured HbA_1c_ in erythrocytes. Therefore, they recommended its use in patients with well‐controlled DM only.^19^ Likewise, another study restricted its use among patients with good glycaemic control.^7^ Thus, the mathematical formula cannot be used interchangeably with measured HbA_1c_ levels.^19^ Using the calculated HbA_1c_ was justified by concern regarding checking HbA_1c_ every three months may be premature to evaluate the effect of basal insulin if the fasting blood glucose has not achieved the goal for two to three months.^18^ Moreover, HbA_1c_ reflects the last 120‐day average, which lags behind the current improvement in glycaemic control.^18^ In addition, the mathematical model used to calculate HbA_1c_ by utilizing measured fasting plasma glucose levels provides the ability to monitor intermittent HbA_1c_ levels between scheduled check‐up visits in patients on anti‐diabetic therapy.^4^ It can be used like the estimated average glucose for chronic glycaemia and acute glycaemia, as well as offer patients a better understanding of current glycaemic control based on daily glucose measurements.^20^ Our findings of a significant difference between the calculated and measured HbA_1c_ levels might be explained by the significant correlation between the FBG and HbA_1c_, which has been documented in recent studies.^21^, ^22^, ^23^, ^24^ Furthermore, no sex differences were observed in a linear relation between glucose and HbA_1c_ among Greek male and female patients with DM.^25^ Interestingly, FBG was strongly correlated in a group of Japanese patients with uncontrolled type 2 DM (HbA_1c_ > 8.0%).^26^ This was in accordance with the observation that higher blood sugar levels predicted higher levels of HbA_1c_ among patients with a pre‐diabetic range in Indonesia.^24^ A significant correlation between HbA_1c_ and FBG levels was reported among patients with and without DM.^27^ In Zambia, it has been observed that there was correlation between HbA_1c_ and FBG.^28^ Hence, they recommended FBG as a suitable and alternative tool to assess glycaemic control in the absence of HbA_1c_.^28^ The availability of facilities to conduct regular checks for HbA_1c_, cost issues and poor health insurance was among the factors in favour of using the calculated HbA_1c_.^5^, ^8^ Moreover, the adopted formula was simple, faster, more cost effective and just as reliable as the chemical analyses.^4^ In contrast, the measured HbA_1c_ test is relatively expensive, so inexpensive alternative testing methods that can be converted to HbA_1c_ are definitively helpful. It is worth to be mentioned that different methods which were used to measure HbA_1c_ showed acceptable accuracy.^29^

## 5 CONCLUSIONS

There was no agreement between the calculated and measured HbA_1c_ levels despite being significantly correlated. Hence, the calculated HbA_1c_, based on the adopted equation, was not a reliable test in assessing glycaemic control among patients with type 2 DM in this study.

### 5.1 Limitation of the study

Other factors that could have effect on HbA_1c_ such as ferritin level and inflammatory markers were not assessed. Other factors which were reported to influence the control of DM, for example vitamin D level were not investigated.^30^

## CONFLICT OF INTERESTS

The authors declare that they have no competing interests.

## AUTHOR CONTRIBUTIONS

Imad R Musa and Ishag Adam involved in conceptualization. Saeed M Omar, Manal E Sharif and Abdel B A Ahmed involved in methodology. Imad R Musa, Saeed M Omar and Ishag Adam involved in data curation. Manal E Sharif, Abdel B A Ahmed and Ishag Adam involved in formal analysis. Manal E Sharif and Abdel B A Ahmed involved in investigation. Imad R Musa, Saeed M Omar, Manal E Sharif, Abdel B A Ahmed and Ishag Adam involved in writing‐original draft preparation.

## Data Availability

The data sets used and/or analysed during the current study are available. From the corresponding author on reasonable request.
